# Boosting Action Observation and Motor Imagery to Promote Plasticity and Learning

**DOI:** 10.1155/2018/8625861

**Published:** 2018-11-07

**Authors:** Ambra Bisio, Michela Bassolino, Thierry Pozzo, Nicole Wenderoth

**Affiliations:** ^1^Department of Experimental Medicine, Section of Human Physiology, Centro Polifunzionale di Scienze Motorie, University of Genoa, Genoa, Italy; ^2^Center for Neuroprosthetics, School of Life Science, Swiss Federal Institute of Technology (Ecole Polytechnique Fédérale de Lausanne), Campus Biotech, Geneva and Campus SUVA, Sion, Switzerland; ^3^Laboratory of Cognitive Neuroscience, Brain Mind Institute, School of Life Science, Swiss Federal Institute of Technology (Ecole Polytechnique Fédérale de Lausanne), Campus Biotech, Geneva, Switzerland; ^4^INSERM, U1093, Cognition Action Plasticité Sensorimotrice, Université de Bourgogne, Dijon, France; ^5^Fondazione Istituto Italiano di Tecnologia, Centro di Neurofisiologia Traslazionale c/o Sezione Fisiologia Umana, Ferrara, Italy; ^6^Neural Control of Movement Laboratory, ETH Zurich, Zurich, Switzerland

In a continuum from fundamental to applied research, many significant scientific contributions in interdisciplinary research fields such as cognitive neuroscience, sport science, and neurorehabilitation provided convincing evidence that action observation (AO, the process of observing actions performed by other people) and motor imagery (MI, the mental execution of action without an overt motor output) might enhance the efficacy of motor training and/or motor recovery by stimulating the activity of the sensorimotor system [1, 2]. The scientific rationale behind this idea is that AO and MI activate neural substrates partially overlapped with those activated by movement execution [3–5]. The existence of a shared neural representation would support the hypothesis that AO and MI may promote neural plastic changes and behavioral improvements in a way similar to movement execution. Moreover, a growing body of evidence in healthy adults proposed that the combination of AO and MI with each other [6] or with central and peripheral noninvasive stimulations might have a greater impact on brain plasticity and motor learning than when these techniques are applied alone [7–10].

In line with this emerging hypothesis, this special issue was published. Authors from 11 countries across Europe, Asia, America, and Australia submitted scientific papers in the format of research article (13), clinical study (2), and review article (2) proposing interesting new insights or reviewing the literature on this topic in different research fields such as neurophysiology, human neuroscience, rehabilitation, and sport neuroscience. The result is a collection of 17 articles showing an increasingly widespread interest in studying the neural mechanisms underlying AO and MI and in applying them in combination with movement execution or other stimulation methodologies.

Four neurophysiological studies examined the combination of MI and AO with peripheral and central stimulations and motor practice to access whether and how these combined techniques evoked changes to the central nervous system activity and improvements in behavioral tasks. In particular, E. Traverse at al. investigated how MI associated with somatosensory electrical stimulation (SS) modulated corticospinal and spinal excitability with respect to MI and SS applied alone. The study by E. Saruco et al. addressed the timing-dependent effects of MI combined with anodal transcranial direct current stimulation (a-tDCS) on improving the performance during a postural task. A critical view on the efficacy of AO associated with a-tDCS in providing advantages to motor learning was raised by the results presented by D. Apšvalka et al. who investigated whether a-tDCS applied over the primary motor cortex during observational practice facilitated the acquisition and retention of a keypress sequence learning task compared to a sham treatment. Doubts on the efficacy of observational practice, when compared to physical practice, were expressed in the neurophysiological and behavioral investigation by N. Alhajri et al., which evaluated the degree of mu suppression in those conditions.

AO and MI also represent valuable tools to investigate how brain activity changes as a function of age, pathological conditions, or motor expertise. A. Mouthon et al. examined age-related differences in cortical and subcortical activities during AO and MI of postural tasks in an fMRI study. Another fMRI investigation (L. P. Kirsch et al.) evaluated how learning a complex motor skill through physical and observational practices shapes neural and behavioural responses among a dance-naïve sample of young and elderly adults. Current theories on the mechanisms underpinning mirror neuron system (MNS) activation during AO and mirror visual feedback (MVF) in stroke are reported in the review article by J. J. Q. Zhang et al. Related to this topic is the research article from F. Bähr et al. in healthy participants, which clarified that although video therapy and MVF applied separately improved the motor performance, video therapy + MVF had no additional boosting effect. Changes in reciprocal inhibition of the forearm during kinesthetic MI and after an MI-based brain-machine interface training were assessed by M. Kawakami et al. in stroke patients. The functional connectivity networks formed on the sensorimotor cortex were measured by means of EEG recordings in subjects with incomplete spinal cord injury and healthy controls by A. Athanasiou et al. during a task combining AO and visual MI simultaneously. The functional difference between visual MI perspectives, namely, internal/kinesthetic vs. external/visual, was investigated using a mental chronometry paradigm by S. Montuori et al. in healthy participants with different levels of motor expertise in pilates with the aim of offering new insights into the application of mental training techniques in sport. Finally, a review paper from Kuehn and Pleger extends the effect of AO on the tactile domain, by summarizing studies assessing the role of visual cues related to the body or to the observation of touch in boosting tactile processing and promoting somatosensory plasticity.

Five original studies offer new evidence on the efficacy of combining conventional rehabilitation techniques with AO therapy (AOT) and MI practice to promote functional recovery in neurological and orthopedic patients. The efficacy of AOT applied during the rehabilitation of upper limb motor functions in children with cerebral palsy was assessed in the study by G. Buccino et al. through a behavioral paradigm based on clinical scales and an fMRI investigation. E. Pelosin et al. tested the effects of an AOT program delivered in a group-based setting compared with standard physical therapy in improving freezing of gait episodes and mobility in subjects with Parkinson's disease (PD). Improvements in mental imagery ability, disease severity, motor and cognitive functions were the ambitious aims of the Dynamic Neuro-Cognitive Imagery (DNI) training administered to PD patients by A. Abraham et al. A proof of concept by E. Durand et al. evaluated the efficacy of personalized observation, execution, and mental imagery (POEM) therapy, a new approach designed to integrate sensorimotor and language-based strategies to treat verb anomia. Although AO and MI are cognitive stimulation methodologies mostly applied during neurorehabilitation, their efficacy was also shown in the treatment of orthopedic patients. U. Marusic et al. administered an AO + MI intervention combined with conventional rehabilitation techniques to verify its effectiveness in older adults after total hip arthroplasty.

In conclusion, this special issue is aimed at providing a fresh state of the art about new means to evoke neural plasticity and behavioral improvements in healthy adults and sportsmen and suggests innovative therapeutic approaches in addition to pharmacological and conventional treatments during rehabilitation. This collection of papers, together with the existing literature, prompts the use of AO and MI in combination with other stimulation techniques and/or motor practice as a valuable research tool for investigating brain physiology in healthy and pathological conditions and as a fruitful intervention methodology to cope with behavioral and cognitive deficits. Although further studies are necessary to elucidate the brain mechanisms underlying these combined stimulation techniques and to address the criticisms also discussed in this special issue in order to improve and rationalize such a tool, the potentiality of these methods is promising for both clinical and sport performance applications. We hope that this special issue will encourage scientists from different domains to deeply investigate how to boost AO and MI effectiveness with the final aim of discovering new tools for rehabilitation and for performance enhancements in sports.
